# Retraction notice to "Treatment of postoperative emetic symptoms with granisetron in women undergoing abdominal hysterectomy: A randomized, double-blind, placebo-controlled, dose-ranging study" [CTR 65/4 (2004) 321-329]

**DOI:** 10.1016/j.curtheres.2018.03.001

**Published:** 2018-03-26

**Authors:** Yoshitaka Fujii, Hiroyoshi Tanaka, Yoshiaki Somekawa

**Affiliations:** 1 Department of Anesthesiology, University of Tsukuba Institute of Clinical Medicine,Tsukuba, Ibaraki, Japan; 2 Departments of Anesthesiology and Surgery, Toride Kyodo General Hospital, Toride, Ibaraki, Japan

This article has been retracted: please see Elsevier Policy on Article Withdrawal (https://www.elsevier.com/about/our-business/policies/article-withdrawal).

This article has been retracted: please see Elsevier Policy on Article Withdrawal (http://www.elsevier.com/locate/withdrawalpolicy).

The following articles are being retracted as a result of overwhelming evidence of fabrication, related to the fact that the distributions of many variables reported by Dr Fujii in these studies could not have occurred by chance, and the inability of Dr Fujii’s institutions to attest to the integrity of the study and/or its data conducted under their auspices. Because both the Journal’s independent and collaborative attempts have not yielded this required information, the Journal has decided to retract the remaining journal manuscripts authored by Dr. Yoshitaka Fujii. The Publisher apologises that the implementation of this retraction was delayed due to an administrative oversight.

Fujii Y, Tanaka H, Kawasaki T. A randomised, double-blind comparison of granisetron alone and combined with dexamethasone for post-laparoscopic choleystectomy emetic symptoms. Current Therapeutic Research 2003;64:514-21. https://www.sciencedirect.com/science/article/pii/S0011393X03001590

Fujii Y, Tanaka H, Somekawa Y. Treatment of postoperative emetic symptoms with granisetron in women undergoing abdominal hysterectomy: a randomised, double-blind, placebo-controlled, dose-ranging study. Current Therapeutic Research 2004;65:321-9. https://www.sciencedirect.com/science/article/pii/S0011393X04800018

